# N-Acetylcholinesterase-Induced Apoptosis in Alzheimer's Disease

**DOI:** 10.1371/journal.pone.0003108

**Published:** 2008-09-01

**Authors:** Debra Toiber, Amit Berson, David Greenberg, Naomi Melamed-Book, Sophia Diamant, Hermona Soreq

**Affiliations:** 1 Department of Biological Chemistry, The Hebrew University of Jerusalem, Jerusalem, Israel; 2 Interdisciplinary Center for Neuronal Computation (ICNC), The Hebrew University of Jerusalem, Jerusalem, Israel; City of Hope Medical Center, United States of America

## Abstract

**Background:**

Alzheimer's disease (AD) involves loss of cholinergic neurons and Tau protein hyper-phosphorylation. Here, we report that overexpression of an N-terminally extended “synaptic” acetylcholinesterase variant, N-AChE-S is causally involved in both these phenomena.

**Methodology and Principal Findings:**

In transfected primary brain cultures, N-AChE-S induced cell death, morphological impairments and caspase 3 activation. Rapid internalization of fluorescently labeled fasciculin-2 to N-AChE-S transfected cells indicated membranal localization. In cultured cell lines, N-AChE-S transfection activated the Tau kinase GSK3, induced Tau hyper-phosphorylation and caused apoptosis. N-AChE-S-induced cell death was suppressible by inhibiting GSK3 or caspases, by enforced overexpression of the anti-apoptotic Bcl2 proteins, or by AChE inhibition or silencing. Moreover, inherent N-AChE-S was upregulated by stressors inducing protein misfolding and calcium imbalances, both characteristic of AD; and in cortical tissues from AD patients, N-AChE-S overexpression coincides with Tau hyper-phosphorylation.

**Conclusions:**

Together, these findings attribute an apoptogenic role to N-AChE-S and outline a potential value to AChE inhibitor therapeutics in early AD.

## Introduction

In Alzheimer's disease (AD), premature death of cholinergic neurons is associated with accumulation of neurofibrillary tangles, constituting of hyper-phosphorylated Tau [1]. The cholinergic hypothesis attributes the cognitive impairments in AD to the loss of cholinergic functions [2]. Accordingly, acetylcholinesterase (AChE) inhibitors serve to ameliorate symptoms by prolonging acetylcholine (ACh) availability [3]. Some argue for attenuation of the disease process under treatment with AChE inhibitors [4], [5]; others develop alternative AD therapeutics, including inhibitors of the Tau kinase, Glycogen Synthase Kinase 3 (GSK3) [6], [7], or of other key proteins of the apoptotic pathway, but it is still unclear if these different approaches reflect a single targeted cascade and if so, what triggers this cascade.

Apoptotic cell death leads to cortical shrinkage in AD brains, accompanied by massive loss of cholinergic neurons, which express considerably more AChE than other neuron types [8]. Recent reports demonstrated AChE accumulation in apoptotic cells, and AChE inhibition and general silencing were found to prevent apoptosome formation and cell death [9], [10]. Such cell death may occur through activation of the endoplasmic reticulum (ER), mitochondrial stress and/or cell surface death receptors [11], [12]. However, these reports raised a new question: how do AChE-expressing neurons survive? Importantly, AChE is not one but several variants, induced by alternate promoter usage and alternative splicing [13]. It occurred to us that some, but not all, AChE variants, may lead to the neuronal cell death which occurs in AD. To challenge this theory, we studied AChE expression in the AD cortex, tested the effects of aberrant AChE gene expression in cultured cells, explored the molecular mechanism(s) involved by manipulating both AChE and key apoptotic proteins, and searched for pharmacological means capable of mitigating the observed apoptotic effects.

## Results

### N-AChE-S expression induces caspase-mediated cell death

**Figure 1:**
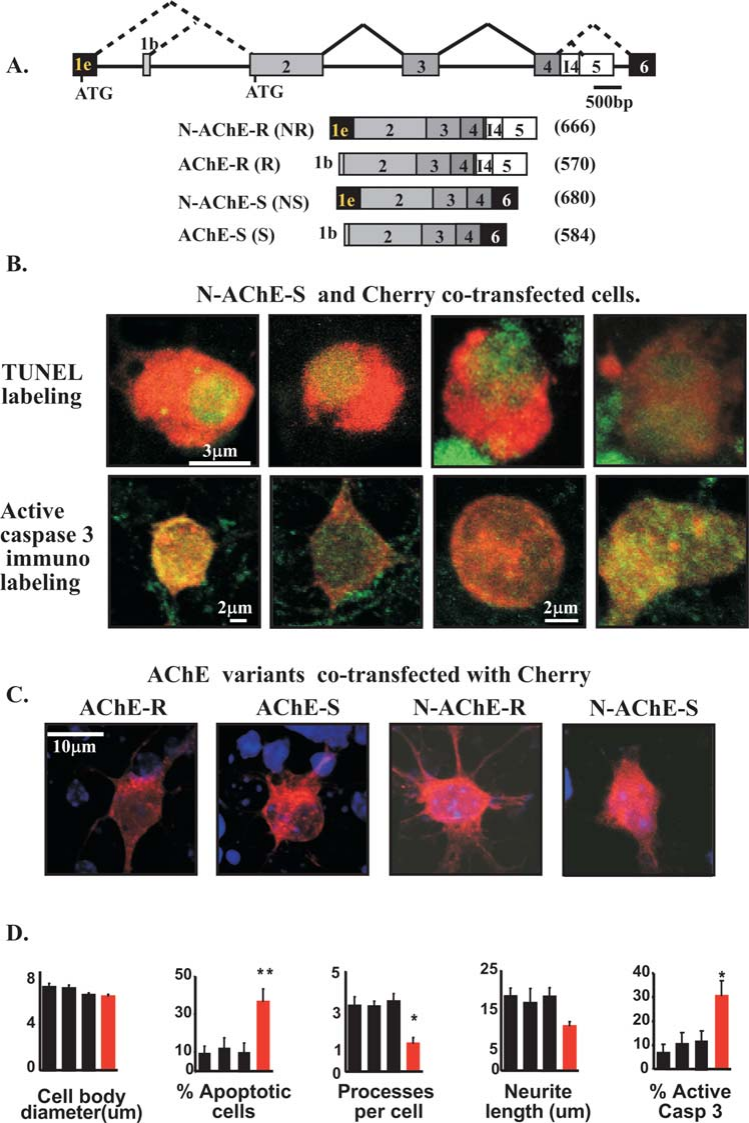
N-AChE-S induced apoptosis in primary cortical cells. A. AChE mRNA transcripts. Upper scheme: The AChE gene structure. Alternate ATG codons are indicated. Lower scheme: corresponding transcripts with respective open reading frames noted (amino acids). B. Primary cortical cells. Upper micrographs: N-AChE-S and Cherry cells co-transfected with (red) were TUNEL labeled (green). Lower micrographs: Red labeling as above, green label shows active caspase-3. C. Effects of AChE variants. Primary cortical cells 24 hr after co-transfection with Cherry and different AChE variants co-transfected with Cherry show non apoptotic cells with different characteristics. N-AChE-S transfections confers shrunk features. D. Shown are cell body diameters, percent of apoptotic cortical cells, No. of ramifications extended from cell bodies, average neurites length, and percent of cells labeled with caspase 3 activated antibody for cells transfected with (from left to right) AChE-R, AChE-S, N-AChE-R and N-AChE-S (red columns). Note N-AChE-S induced changes(*p = 0.001,**p = 0.0001 Student's t test).

**Figure 2:**
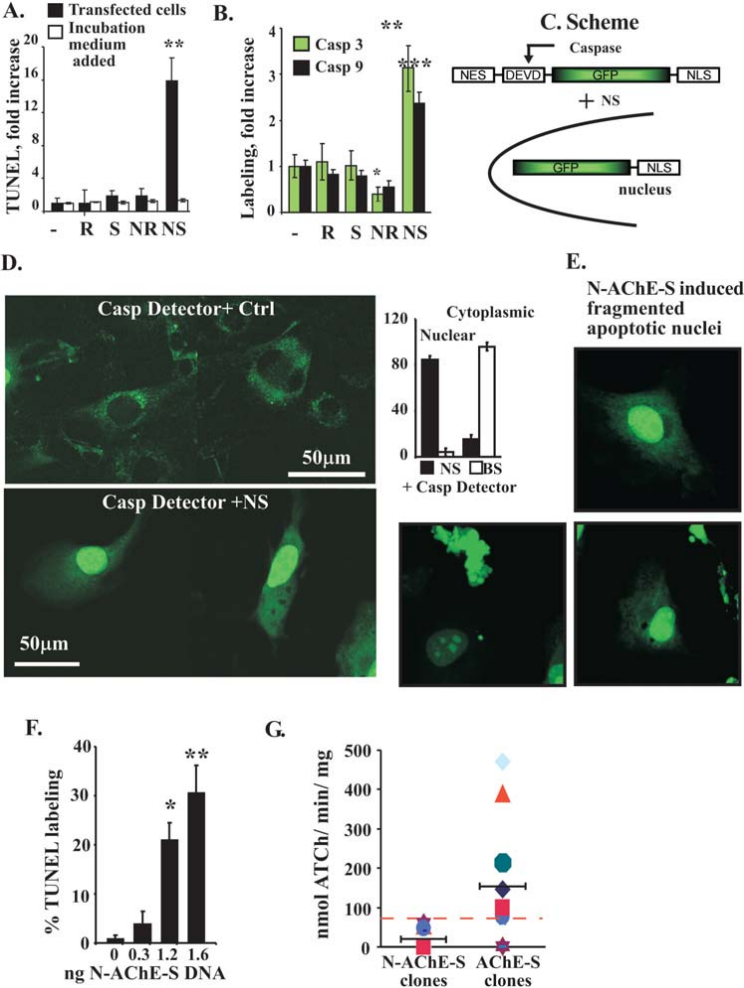
N-AChE-S induces caspase-mediated cell death. A. N-AChE-S mediated apoptosis. TUNEL analysis 24 hr post-transfection in U87MG cells transfected with AChE variants or an “empty” plasmid, compared with non-transfected cells incubated with pre-conditioned medium (*p = 0.001, Student's t test). B. Caspase immunostaining. N-AChE-S-associated increases in activated caspase 3 and 9 and N-AChE-R-associated reduction in caspase 3 in U87MG cells (*p = 0.04, **p = 0.004, ***p = 0.0003 Student's t test). C. Caspase activation reporter. N-AChE-S was co-injected with GFP-NLS vector equipped with NES sequence and a caspase-cleavable DEVD motif. Caspase activation yields nuclear GFP translocation. D. Microinjection-induced caspase-mediated cell death. Micrographs: 3T3 cells co-injected with an “empty” plasmid and GFP-NES/NLS (top micrograph) showed cytoplasmic GFP, whereas N-AChE-S co-injected cells (bottom micrograph) showed nuclear fluorescence (columns). Shown are representative cells, out of N = 70, p<10^−7^ Student's t test. E. Within 24 hr from injection, cells expressing N-AChE-S acquired nuclei with condensed chromatin. F. N-AChE-S-inducible apoptosis is dose-dependent. Percent of apoptotic U87MG cells under transient transfection with increasing plasmid doses. (*p = 0.03, **p = 0.002 Student's t test). G. Effects of stable transfection on cell viability. CHO cells were co-transfected with either N-AChE-S or AChE-S vectors, and the G-418 antibiotic resistance plasmid, and were grown for 14 days in the presence of G-418. Individual surviving clones were isolated and expanded and cellular AChE activity was measured (p = 0.02, Student's t-test). Note that individual clones transfected with N-AChE-S all showed considerably lower activity, largely below the average activity in human brain extracts (dashed line).

Overexpression of two short (AChE-S, AChE-R) and two N-terminally extended AChE variants(N-AChE-S, N-AChE-R) [13] (Fig 1A), was induced by transient transfection of mouse primary cortical cells, HEK 293 embryonic kidney cells, U87MG glioblastoma, T84 lung epithel and CHO hamster ovary cells. In primary cortical cells expressing N-AChE-S, this invariably caused apoptosis, observed as increased TUNEL labeling and caspase 3 activation (Fig 1B and C, respectively). Surviving cortical cells transfected with N-AChE-S showed similar cell body size to those expressing the other variants; however, they extended fewer and shorter processes from the cell body than cells expressing any of the other variants, indicating ill health for transfected surviving cells (Fig 1D). Importantly, no other tested AChE variant exerted such effects (Fig 1E and Table S1).We excluded the possibility of indirect effects of secreted AChE or other proteins, by demonstrating that pre-conditioned medium, removed 24 hr after transfection and added to non-transfected cells, caused no apoptotic effect (Fig 2A and Table S1). Together, this attributed the caused cell death to intracellular overexpression of N-AChE-S. Moreover, N-AChE-S overexpressing cells showed concurrent increases in both activated caspase 3 and 9 (Fig 2B) and the caspase inhibitor Z-VAD-FMK prevented the N-AChE-S induced cell death (see below), suggesting a caspase-mediated apoptotic cascade [14]. Highlighting the specificity of the N-AChE-S-induced effects, caspase 3 levels were inversely reduced in N-AChE-R-transfected cells (Fig 2B).Therefore, the N-terminal extension by itself appeared to be insufficient to cause the cell death conferred by N-AChE-S.

To directly estimate the efficiency and dynamics of the N-AChE-S-induced cell death, we selected 3T3 cells for microinjecting N-AChE-S. Co-injection of a cleavable caspase-activated GFP reporter, equipped with a nuclear localization signal which enables translocation to the nucleus in the presence of activated caspases (Fig 2C, scheme) served for follow-up of the induced changes. The caspase reporter and an “empty” vector were microinjected for control (Fig 2D,E). Within 24 hrs, injection of the caspase reporter alone induced cytoplasmic GFP fluorescence in 94±3% of the cells (Fig 2D). In contrast, 85±3% of the N-AChE-S injected cells showed nuclear GFP fluorescence, reflecting caspase activation, and acquired condensed nuclei characteristic of apoptosis (Fig 2D,E).

In cells transiently transfected with >1.2 ng/cell of the N-AChE-S DNA, N-AChE-S induced dose-dependent apoptosis (Fig 2F). Next, individual clones of CHO cells, which do not express any endogenous AChE variant were stably transfected with N-AChE-S or the shorter AChE-S, for comparison. Cloned cells presented considerably less N-AChE-S protein than clones stably transfected with AChE-S (also called “tailed” [15]). This was reflected in considerably lower ACh hydrolytic activity than that of cells stably transfected with AChE-S (10±8.4 vs. 150±45 nmol substrate hydrolyzed/min/mg protein, Fig 2G, dashed line). Such low activities, lower than the average activity observed in the human brain, indicated a possible selective pressure against expression of N-AChE-S in high levels. Thus, microinjection as well as transient and stable transfections all suggested that excess N-AChE-S is incompatible with cell viability.

### N-AChE-S displays similar catalytic characteristics to other AChE variants

Fractionation and activity tests demonstrated that AChE-S and N-AChE-S largely remained within the cells, whereas AChE-R and N-AChE-R both showed a tendency to be secreted, (Fig S1A).Sequence analysis predicted that while the AChE signal peptide is likely masked by the N-extended terminus, the new N-AChE variants include an additional signal peptide. Higher expression levels for the shorter variants were observed by immunoblot analysis of transiently transfected cells. Enzymatic Activity (with acetyl-thiocholine) staining of native gels showed matched differences in enzyme activity (Fig S1B), indicating proportional expression and activity of the various AChE proteins. Nevertheless, the key enzymatic characteristics (e.g. K*m*, substrate inhibition) were indistinguishable for N-AChE-S and N-AChE-R as compared with the shorter variants (Fig S1C,D), indicating that the N-extended variants are correctly folded. Thus, N-AChE-S was found to be expressed at lower levels than the shorter variants, but despite this, it was the only variant with an apoptogenic effect.

### N-ACHE-S promotes GSK3 activation and Tau hyper-phosphorylation

**Figure 3:**
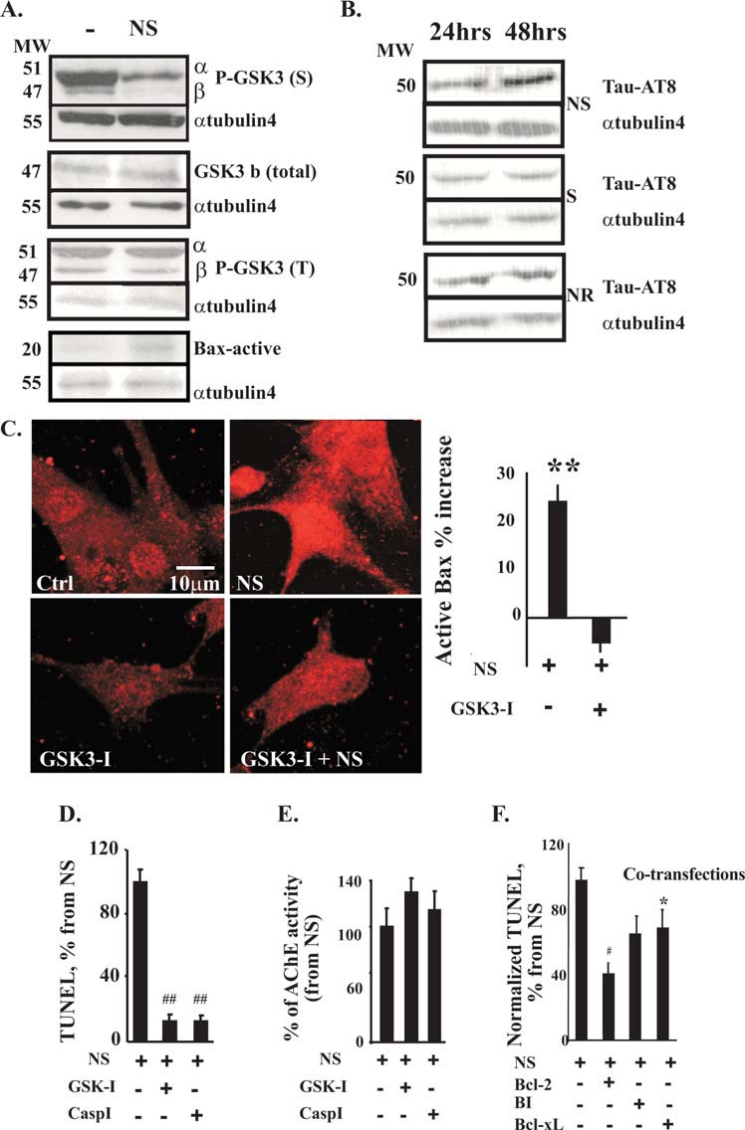
N-AChE-S induces suppressible GSK3-activation, Tau hyper-phosphorylation and Bax activation. A. Immunoblots. N-AChE-S reduces serine-phosphorylated P-GSK3 while maintaining unchanged total GSK3β and tyrosine phosophorylated GSK3, and elevating active-Bax. B. Tau hyper-phoshorylation: N-AChE-S, but neither AChE-S nor N-AChE-R induces progressive increases in hyper-phosphorylated Tau (Tau AT8). Alpha tubulin 4 served as reference. C. N-AChE-S-inducible GSK3 dephosphorylation facilitates Bax activation. Shown are micrographs of N-AChE-S transfected cells with elevated phosphorylated Bax labeling compared to U87MG control (Ctrl) cells. GSK3 inhibition suppresses this labeling. Quantification: % increase in Bax labeling of NS overexpressing cells, preventable under GSK-I (SB316763) treatment (**p = 0.00009 Student's t test). D. GSK3 and Caspase inhibition suppress N-AChE-S-induced apoptosis. % TUNEL labeling of N-AChE-S transfected cells. Note that 5 µM SB316763 (GSK3-I) and 80 µM Z-VAD-FMK (caspase-I) reduced apoptosis. (GSK-I p = 0.000013, Casp3-I p = 9×10^−11^ Student's t test). E. GSK3 and Caspase inhibitors do not affect AChE catalytic activity. F. Bcl proteins suppress N-AChE-S-induced apoptosis: U87MG cells co-transfected with N-AChE-S and Bcl-2 (# p = 0.00001), BI (*p = 0.03), or Bcl-XL (p = 0.054 Student's t test) showed variably suppressed TUNEL labeling.

The relatively low levels of N-AChE-S could potentially be due to misfolded proteins activating ER stress-induced apoptosis. To test this possibility, we measured the levels of the ER stress markers GRP78 and Xbp. Both GRP78 levels and the ratio between spliced and unspliced variants of Xbp were similar to those of cells transfected with the other variants or control cells (Fig S2A,B), suggesting that the observed apoptogenic effects were not caused by the misfolded protein response or the ACh hydrolytic properties of N-AChE-S. Nevertheless, high levels of N-AChE-S could signal the cell of a “possible threat”, inducing another kind of cellular stress, compatible with other cases where an overexpressed protein can lead to cell death, e.g. hIAPP in B cells[16]. Next, we explored relevance to the observed apoptosis of the Tau kinase GSK3, which regulates apoptosis-modulating Bcl proteins including Bax [17], [18] and was implicated in Alzheimer's disease pathology [19]. We tested the potential involvement of GSK3 in N-AChE-S-induced apoptosis in U87MG cells, which are more readily amenable for transfection than primary neurons. Supporting such involvement, N-AChE-S expression selectively decreased the levels of inactive GSK3 carrying phosphorylated serine, whereas total GSK3β and the levels of hyper-activated GSK3α and β with phosphorylated tyrosine remained unchanged (Fig 3A). Thus, larger GSK3 fractions were active under N-AChE-S expression. Moreover, N-ACHE-S overexpression induced progressive increases in the hyper-phosphorylation of Tau in cultured U87MG cells, reaching a 37% increase by 48 hr post-transfection (P<0.05, Fig 3B). Correspondingly, U87MG cells transfected with N-AChE-S showed 25% enhanced immunolabeling of activated Bax (Fig 3C), susceptible to blockade by the GSK3 inhibitor SB316763, known to suppress cell death [18]. Importantly, SB316763 reduced the immunolabeling of both activated Bax and hyper-phosphorylated Tau in cultured cells by about 20% as compared to N-AChE-S transfected cells with no inhibitors (Fig 3D and data not shown). Also, SB316763 largely prevented the N-AChE-S apoptotic effect, to a similar extent as caspase inhibition, and without affecting AChE's catalytic activity (Fig 3D,E).

### Bcl proteins mitigate the N-AChE-S induced apoptosis

Since AChE silencing was shown to prevent apoptosome formation [9], we tested if the N-AChE-S effect occurs upstream to the mitochondrial membrane permeablization, characteristic of apoptosis. Cultured cells were co-transfected with N-AChE-S and the anti-apoptotic protein Bcl-2 or its family members, Bcl-xL and BI. All of these proteins disable Bax from triggering the permeabilization of mitochondrial membranes, and all suppressed the N-AChE-S-induced cell death (Fig 3F), compatible with the notion of a “classical” apoptotic pathway.

### Cell surface N-AChE-S location

**Figure 4:**
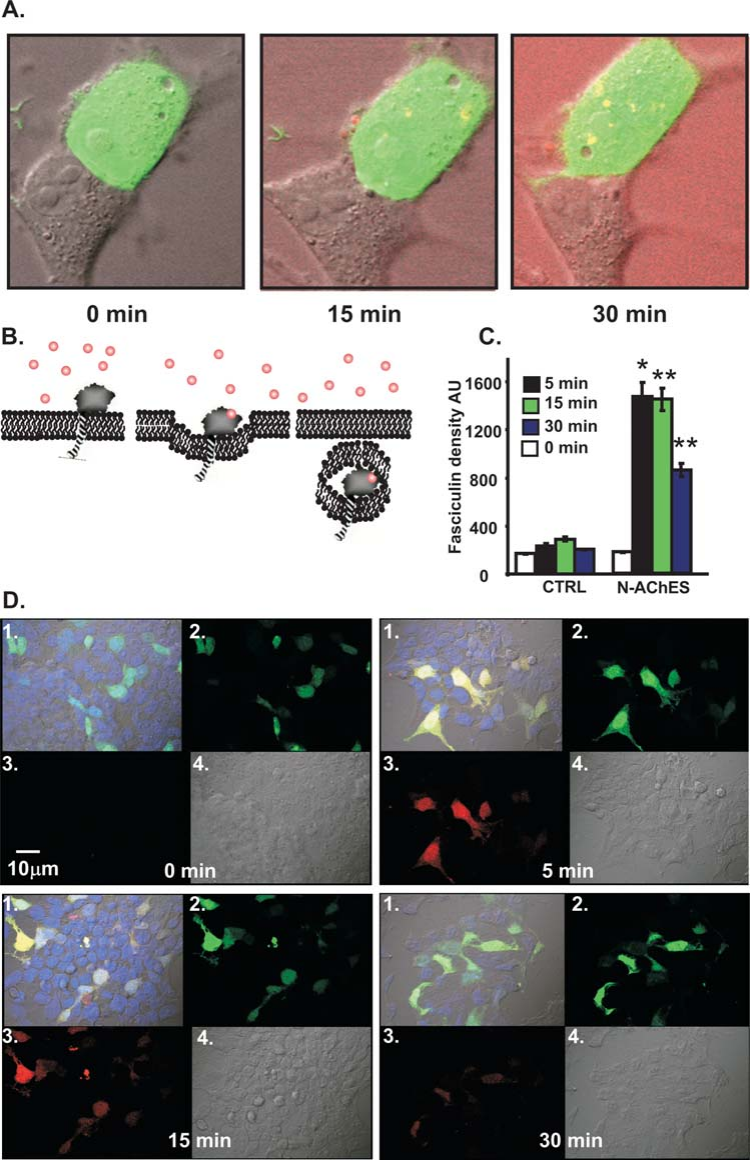
N-AChE-S Membranal localization and Fasciculin-2 Internalization. A. Fasciculin-2 internalization. Tetramethylrodhamine labeled Fasciculin2 was added to the medium of 293 cells co-transfected with N-AChE-S and GFP. Shown is internalization of labeled Fasciculin 2 yielding fluorescence into N-AChE-S transfected, but not non-transfected cells at 15 and 30 minutes. Pictures were taken by live imaging. B. Schematic representation of N-AChE-S in the cell membrane and its internalization. C. Tetramethylrodhamine labeled Fasciculin2 was add to the medium of N-AChE-S and GFP co-transfected 293 cells were fixed after: 0,5,15 and 30 min. The density of Fasciculin2 was measured. Columns show density in N-AChE-S transfected vs non-transfected cells at the noted times (*p>0.05, **p>0.005 Student's t test). D. Representative pictures of the experiment quantifies in C.1.Merge picture (yellow fluorescence notes Fasciculin2 internalization into GFP-expressing cells). 2. Co-transfected GFP and N-AChE-S cells. 3. Fasciculin-2 internalized 4.Light picture.

We considered the possibility that in the N-extended AChE variants, the retained original signal peptide can be used as transmembranal domain similar to the homologous protein neuroligin [20]. To address this issue, we labeled Fasciculin-2, an AChE inhibitor which is impermeable to the cell membrane, with tetramethyl-rodhamine (Fas2*)[21]. 293HEK cells which do not express endogenous AChE were co-transfected with N-AChE-S and GFP for 18 hrs and were exposed to Fas2*. Live imaging was performed before Fas2* addition, and every 5 minutes after its addition. Cells co-transfected with GFP+N-AChE-S but not non-transfected neighbor cells showed vesicular internalization of Fas2*(Fig 4A,B). We than performed the same experiment and fixed cells after 0,5,15 or 30 minutes. Fixation disrupted the vesicles; however, red labeling reflecting internalized Fas2* remained in the cytoplasm of cells expressing GFP+N-AChE-S compared to control cells (Fig 4C,D). Labeling peaked after 5 minutes from Fas2* addition, remained unchanged after 15 minutes and declined after 30 minutes. Our results indicate that N-AChE-S is positioned in the membrane with the catalytic domain towards the extracellular space, allowing Fas2* binding. In addition, we used antibodies specific for the extended N-terminus [22] in comparison to antibodies targeted to the core domain to label N-AChE-S in transfected U87MG cells. An “empty” vector served as control (Fig S3A, scheme). In transmission electron microscopy, N-ACHE-S-transfected cells yielded generally similar labeling patterns with the N-specific and core domain antibodies, peaking up to 6 nm from the cellular membrane (Fig S3A,B). Apart from the membrane-associated labeling, a total of 11% of the gold beads adhered to endocytotic vesicles (identified by their clathrin-coated contours [23], compatible with the Fas2* data.

### Core domain contributions to N-AChE-S-induced apoptosis

**Figure 5:**
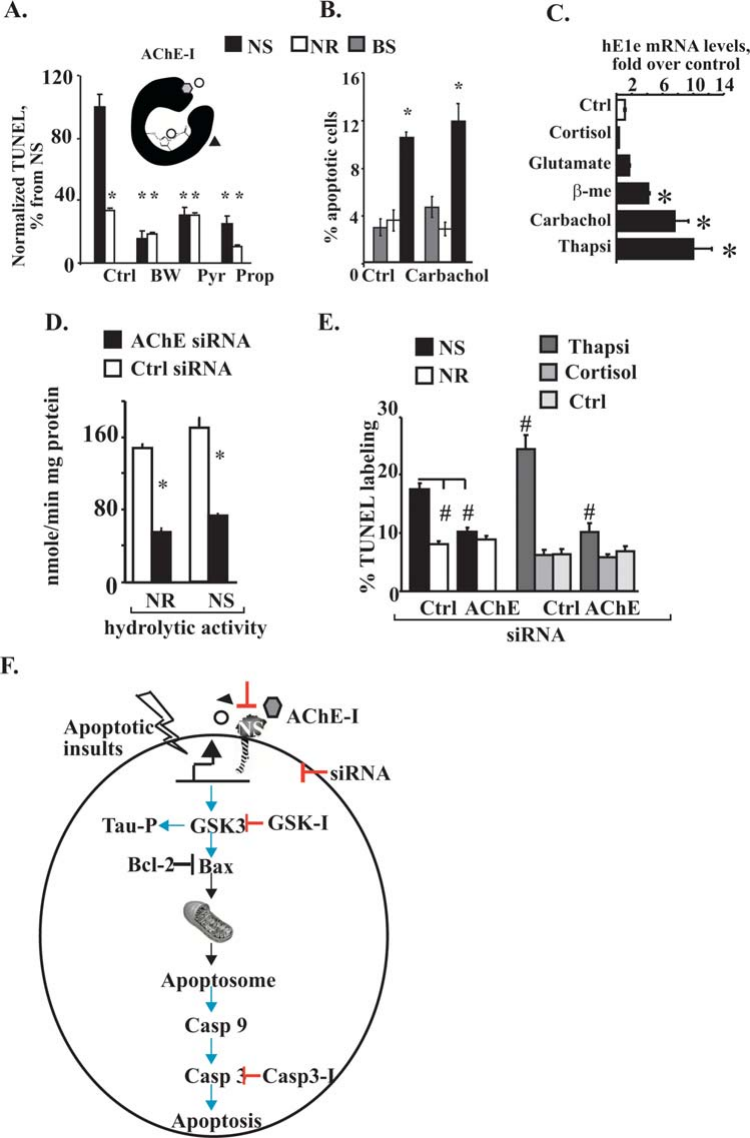
N-AChE-S and Thapsigargin induced apoptosis are suppressed by AChE-I and siRNA. A. Small molecule-mediated suppression of N-AChE-S-induced apoptosis: Normalized TUNEL labeling. Inset drawing: Pyridostigmine (Pyr), propidium (Pro), or BW284C51 (BW) are variably targeted AChE-Is. Columns: All AChE-Is prevented cell death in N-AChE-S but not N-AChE-R-transfected cells (NS vs. NR p = 0.00002, NS vs. NS+Pyr 0.008, NS vs. NS+BW p = 0.000001, NS vs. NS+Pro p = 0.000002 Student's t test) B. Carbachol fails to prevent N-AChE-S-induced cell death. Carbachol addition did not affect cell death in control, N-AChE-R or N-AChE-S (Blue Script, BS is used as empty vector) transfected cells. C. Apoptotic stimuli induce hE1e over-expression. QRT-PCR of U87MG cells subjected to the noted agents. Columns: fold change of hE1e levels over naive cells (Thapsi *p = 0.03, Carb p = 0.03, ß-me p = 0.02 Median test n = 4). D. AChE-targeted siRNA suppression. AChE targeted siRNA but not a control siRNA reduced AChE activity in cells transfected with either N-AChE-S or N-AChE-R, (NR ctrl vs NR siRNA p = 0.002; NS ctrl vs NS siRNA p = 0.002; n = 3 different experiments, Median test). E. siRNA suppression of N-AChE-S-induced apoptosis. Apoptosis was effectively suppressed in cells co-transfected with N-AChE-S and siRNA, or cells treated with Thapsigargin, but siRNA did not affect cortisol-treated or N-AChE-R transfected cells. (NS ctrl vs. NS siRNA p = 0.00009, NS vs. NR p = 0.00002, thapsi ctrl vs. thapsi siRNA p = 0.00001, thapsi vs. ctrl p = 3^−7^ thapsi siRNA vs. crtl siRNA p = 0.033, Student's t test). F. The N-AChE-S apoptotic cascade. NS upregulation by apoptotic insults co-activates GSK3 by serine dephosphorylation, facilitating activation of Bax and caspases, yielding apoptosis preventable by Bcl-2 proteins or GSK3, AChE, and caspase inhibitors.

At the extracellular domain of synaptic membranes, neurexin interactions with neuroligin transduce intracellular signals through synaptic protein partners [20]. In view of our Fas2* and electron microscopy results, we speculated that the intracellular N-terminus of N-AChE-S might similarly mediate intracellular signaling in response to extracellular cues. Given that inhibitor interactions induce structural changes in the AChE core domain [24], we assessed the capacity of the carbamate active site inhibitor, pyridostigmine, the peripheral site inhibitor of AChE, propidium or the quaternary AChE inhibitor, BW284C51, known to be impermeable to the cell membrane [25] to affect N-AChE-S-induced apoptosis. All of these agents reduced apoptosis in N-AChE-S-transfected cells to the levels observed in N-AChE-R transfected or control cells (Fig 5A). These findings demonstrated inherent involvement of the core domain of AChE, where the active site is embedded, in the N-AChE-S-induced apoptotic process. The simplest explanation for the mechanism(s) involved therefore suggests that the N-AChE-S-induced apoptosis may initiate extracellularly, as it is susceptible for suppression by small molecule inhibitors excluded from cellular entrance (e.g. BW284C51) and since such inhibitors appear to prevent the transduction of the signal into the cell.

Since N-AChE-S is enzymatically active, we further wished to test the possibility that cells were dying because of the lack of ACh. Addition of the non-degradable ACh-analog carbachol did not change the observed cell death (Fig 5B), excluding this option and suggesting structural, rather than enzymatic involvement of the core domain in the induced cell death.

### Insult stimuli facilitate inherent N-AChE-S expression and apoptosis

Notably, various cellular and organismal stimuli induce AChE overexpression, alternative splicing and alternate promoter choices [13], [26], [27]. Therefore, we tested the effects of stressors-induced gain of N-AChE-S function on apoptosis outcome. Carbachol (10 µM), ß–mercaptoethanol, a reducing agent which denatures proteins by reducing disulfide bonds (0.2 M), or thapsigargin, which releases calcium from the ER and intracellular stores (10 µM) [28] all induced significant inherent overexpression of the hE1e exon encoding the extended N-terminus in U87MG cells (median test p<0.03). In contrast, glutamate, the NMDA receptor agonist (0.5 mM), and cortisol, a glucocorticoid hormone protecting nerve cells from acute stress responses (0.5 µM) [29] showed no effect on hE1e expression (Fig 5C). This called for testing the consequences of loss of N-AChE-S function on apoptosis. In cells co-transfected with N-AChE-S and an AChE-targeted siRNA, loss of N-AChE-S expression selectively abolished its apoptotic effect. siRNA also ameliorated the cell death induced by thapsigargin; in comparison, suppressed N-AChE-R expression or cortisol treatment had no effect (Fig 5D,E). Thus, inherent gain of N-AChE-S function induced apoptosis, whereas loss of its function suppressed it. Together, these data identified N-AChE-S as an upstream apoptotic trigger encompassing most of the “classical” cell death elements (Fig 5F) and likely acting through the interaction of yet unknown intracellular element(s) with the N-terminal extended domain.

### N-AChE-S expression in AD

**Figure 6:**
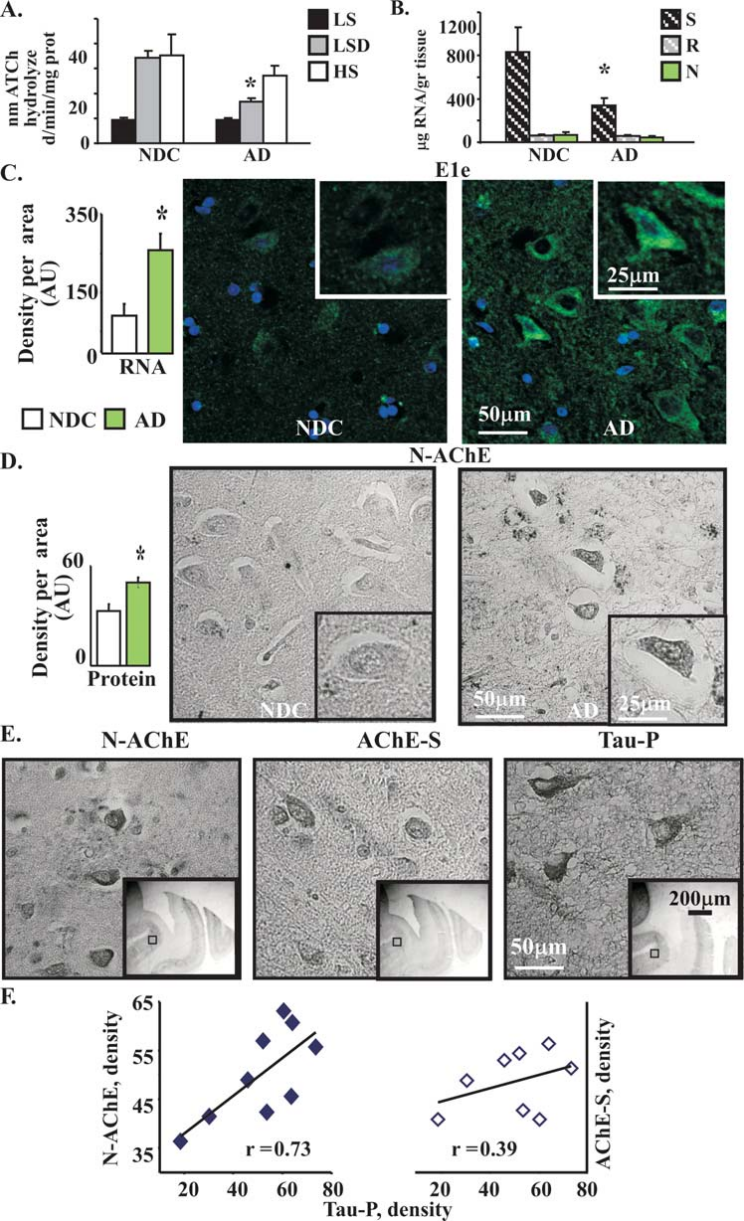
N-AChE-S overexpression in AD cortical neurons associates with Tau hyper-phosphorylation. A. Differential fractionation of AChE hydrolytic activity. Cortical protein was fractionated into low salt (LS, soluble protein), low salt detergent (LSD, membrane-associated) and high salt (HS, integral membrane and nuclear proteins) and AChE activity was measured. Note decrease in LSD (AChE-S-enriched) fraction, but not LS or HS fractions (p = 0.0476, n = 4 NDC, 8AD, Median test). B. AD-associated changes in AChE mRNA transcripts. Columns: QRT-PCR. Note AD-associated decrease as compared to NDC samples in the S, but not the N or R encoding exons. (p = 0.055, n = 10 AD, 5 NDC Wilcoxon Two Sample Test). C. Inset is: E1e labeling of post mortem sections from human entorhinal cortex. Columns are quantification of labeling density per area. (Median test, *p = 0.0286, n = 4 in each group). D. N-AChE-S Immunostaining in AD and NDC cortices. Columns are quantification of density per area (*p = 0.029 n = 5 NDC, 9AD Median Test). E. AD Entorhinal cortices immunolabeled for N-AChE, AChE-S or hyper-phosphorylated Tau-P. F. N-AChE and AChE-S labeling correlate with Tau-P density. Tau-P, N-AChE, and AChE-S labeling were quantified using immunohistochemistry on sections from the entorhinal cortex of AD patients. (Pearson's correlation for N-AChE and Tau-P, 0.73, p = 0.03).

Last, but not least, we turned to the AD brain. When protein from cortical tissues was differentially extracted in detergent and salt-containing solutions, AD cortices predictably showed reduced AChE activity as compared to matched non-demented controls (NDC). Reduction was observed in the membrane-associated fraction (where AChE-S is present [30]) (Fig 6A). Neither the soluble nor the membrane protein fractions showed such decreases, indicating that the reduction was primarily of AChE-S, likely due to the massive loss of cholinergic neurons. In NDC cortices, quantitative real-time RT-PCR calibrated using each cDNA clone showed that transcripts spanning the hE1e exon which encodes the N-terminal extension represent approximately 6% of total AChE mRNA (4000 transcripts per ng RNA, compared to 55,700 transcripts of total AChE mRNA per ng). Parallel tests showed that both the core domain and the major exon 6 decreased by 40+/−26% in AD as compared with NDC cortical tissues (Fig 6B, and data not shown). Therefore, AD tissues contained less AChE-S (which normally constitutes 90% of the enzyme) but similar amounts as NDC of N- AChE and AChE-R (each constituting about 6% of AChE mRNA transcripts).

Our mRNA analyses suggested that in the remaining cholinergic neurons N- AChE-S constitutes a larger part of AChE than in NDC brains. To challenge this hypothesis, we used quantified FISH analysis which indeed identified 273% enhanced hE1e expression in AD cortical cells with neuronal morphology as compared with NDC sections (Median test p = 0.03) (Fig 6C). Immuno-labeling further demonstrated 152% elevated levels of the N-terminus characteristic of N-AChE-S in AD compared to NDC cortices (Median Test p = 0.03) (Fig 6D), together suggesting AD-associated overexpression of N-AChE-S in cortical neurons.

### Association with hyper-phosphorylated Tau in AD

Tau hyper-phosphorylation is a marker of AD pathology that is directly related to cell death. Importantly, N-AChE and AChE-S showed similar patterns of expression to that of hyper-phosphorylated Tau in the AD cortices. The majority (72, 70 and 77% of measured cells out of approximately 100 in each group) of cells expressing N-AChE, AChE-S or hyper-phosphorylated Tau were 7–24 µm in length, compatible with the size of interneurons (Fig 6E). Moreover, the labeling intensity of N-AChE, and to a somewhat smaller extent also of AChE-S (probably because only part of the AChE-S molecules are N-AChE-S) were directly correlated with that of hyper-phosphorylated Tau (Fig 6F). Together with the cell studies, these observations are compatible with the hypothesis that N-AChE-S overexpression in AD cortical neurons is causally linked with the apoptotic destiny reported for neurons expressing hyper-phosphorylated Tau.

## Discussion

Recent reports of AChE association with apoptosis [9], [10] left unexplained the long-term survival of AChE-expressing cholinergic neurons. Our current findings provide an unequivocal answer to this question by pointing at N-AChE-S as the only apoptotic AChE variant. We further demonstrate that N-AChE-S induces cell death only when present above a critical threshold. To explore the molecular mechanism(s) underlying this apoptotic role, and to study its relevance for the AD-induced death of cholinergic neurons, we systematically manipulated N-AChE-S expression in cell culture experiments, and demonstrated that gain of N-AChE-S function invariably induces apoptosis whereas loss of its function improves survival.

Enforced N-AChE-S overexpression induced apoptosis both in primary cortical cells and in different cell lines of various tissue and organismal origins. This attributes to this protein a conserved and ubiquitous role as a general trigger of apoptosis. In the AD cortex, neuronal loss reaches 30–90% [31] in a manner attributable to the formation of hyper-phosphorylated Tau and neurofibrillary tangles [1]. Circulation AChE levels are elevated during normal aging [32]; moreover, the ApoE4 genotype [33], mutations in presenilins [34] and the presence of Aß [35]–[37] all induced upregulation of AChE activity. AChE upregulation was also reported as being induced by the apoptotic JNK pathway [38], as well as by damaging light in retinal neurons [39]. Here we report that acute cellular stress reactions and calcium misregulation, both implicated in AD and in the susceptibility to cell death [40], lead to N-AChE-S overexpression. Our current findings thus indicate that the protein involved in all the above phenomena was likely the N-ACHE-S variant. Further studies will be required to explore the possible function(s) of N-AChE-S in normal tissues, and define if it is involved, for example in the programmed cell death characteristic of developing neurons [41].

The N-AChE-S, but not the N-AChE-R or the shorter AChE-S variants induced cell death, suggesting that both the N- and the-S termini are required for this process. Additionally, various AChE inhibitors suppressed the N-AChE-S induced apoptosis pointing at the central core domain, where AChE's active site is embedded [42] as being causally involved as well. The most likely explanation for this multi-domain involvement is that structural, rather than enzymatic features of N-AChE-S protein are involved. We localized part of the N-AChE-S variant to the cell membrane and demonstrated extracellular inhibitor interactions and rapid internalization for this protein, suggesting that the extended N-terminus of N-AChE-S protrudes intracellularly, similar to the homologous protein neuroligin [20]. Inhibitor interactions were shown by others to exert structural changes on the AChE molecule [43], indicating parallel changes in N-AChE-S. That various AChE inhibitors attenuate the N-AChE-S-induced apoptotic outcome therefore suggests that, the core domain transduces signals to the cell through the cytoplasmic N-terminal domain. Together, this implies that N-AChE-S likely operates as a bifunctional entity having properties characteristic of both enzymes and ligand-activated receptors. Considering the above arguments, these findings may also explain the reported disease-modifying effects of anticholinesterases [4], [5] and can possibly outline testing their future use as prophylactic agents.

Our current finding of increased N-AChE expression in AD cortices, which lose 30–50% of their core AChE expression and activity, is compatible with the enhanced apoptosis in these tissues. Supporting the notion of causal relationship with this process, both cellular stress and calcium imbalances have been implicated in GSK3 activation and in cell death in the AD brain [12] through hyper-phosphorylated Tau, presenilin, APP and Bax [18]. The N-ACHE-S-induced dephosphorylation of the Ser residue activating GSK3 kinase properties corroborates this association. Others reported increased Bax levels and TUNEL staining in neurons with Tau hyper-phosphorylation. A recent report, however, argues that Tau hyper-phosphorylation is protective [44]. Our findings did not distinguish between these possibilities but demonstrate apparent association of Tau hyper-phosphorylation with the N-AChE-S apoptotic mechanism. We further predict that exposure to apoptotic stressors would likely induce N-AChE-S expression, particularly in the cholinergic neurons with higher basal AChE expression, and cause apoptotic cell death. At least part of the currently employed AD therapeutics may ameliorate the effect of this overexpression by preventing the N-AChE-S-induced apoptosis, thus delaying disease progression [4], [5]. Further delineation of this induction and ways to manipulate it for preventing the premature death of cholinergic neurons at earlier stages of the disease would therefore merit special attention.

## Materials and Methods

### Human brain tissue

Brain tissue from AD patients (n = 10) and matched non-demented controls (n = 5) was obtained from The Netherlands Brain Bank. Ethical approval and written informed consent from the donors or the next of kin was obtained in all cases. Neuropathological Braak staging of neurofibrillary changes (I–VI) was performed *post mortem* (Table S2).

### Primary cortical cell culture

Cerebral cortex was separated from the brain of 15^th^ day embryos of FVB/N mice, minced, and cells plated on poly-L-ornithine coated cover slips in Neurobasal medium, B27 supplement, Glutamax and Penicillin/streptomycin (Invitrogen). Transfections were performed with lipofectamine 2000 (Invitrogen).

### Plasmids

The N-AChE-R and N-AChE-S plasmids are modifications of CMV-AChE-S and CMV AChE-R with the hE1e exon inserted in the HindIII cloning sites. Bax Inhibitor Plasmid (BI) was from Science Reagents El Cajon, CA, and the caspase detector was from Clontech (Palo Alto, CA).

### Microinjection

NIH 3T3 cells were microinjected using an Axiovert200M Zeiss microscope equipped with a microinjection system, incubated for 24 hours and then viewed. This involved an MRC-1024 BioRad confocal scanhead coupled to a Zeiss Axiovert 135M inverted microscope with a 40×/NA = 1.3 oil immersion objective. GFP was detected at 488 nM the excitation wavelength and a HQ525±20 filter served for collecting emission.

### Statistics Methods

**Student's t-test** was used in cell culture experiments, where cell numbers were large enough to assume normal distribution.

**Median test** is a-parametric test was used for comparing the median value of two different populations that were relatively small, assuming abnormal distribution (minimum 4 samples, maximum 10 e.g. mRNA transcripts, protein densities and protein activity) as compared to control.

**The Wilcoxon test** was preferred over the median test when one or more observations were “off scale”. It served for calibrating cDNA transcripts from AD and control samples.

**Pearson correlation test** describing the strength of an association between variables was used to measure the correlation between N-AChE-S and hyper-phosphorylated Tau. The aim was to test if *linear* relationship exists between these values of variables.

### Image analysis

Immunohistochemistry and In situ hybridization experiments were quantified by the ImageJ 1.33 free software (http://rsb.info.nih.gov/ij/).

1. In the “Analyze tool” we chose Set measurements and selected: Mean gray value (which measures the density of the selected pixels divided by the number of pixels), to quantify staining intensity.

2. We used the “freehand selection” tool to draw a line around a single cell we wanted to measure and then selected an appropriate measure in the “analyzis” tool; in all cases, we analyzed many cells (20–30) in each picture. We selected 2–3 empty spots in the tissue to determine the background for each picture, and subtracted it from each of the values observed for the measured cells to normalize light efficiency differences that could occur between different pictures.

3. The total average for a single experiment was composed of 4 different pictures at least. Standard deviation values were calculated for these averages, to challenge the possibility that a specific change was an outlier which occurred outside the normal range calculated for the other pictures.

The averages of the different groups were used and are depicted in the figures.

4. Data collected from the different cells in each picture was used to derive an average value for that picture. In case of cell culture experiments, 5–8 different pictures were used to assess each treatment and were analyzed as noted before.

5. For Western Blot analysis we used the ImageJ Gel Analyzer option, in which we marked a rectangular shape for each band in the gel. We captured the graph view of the intensity as area of the band, which should correlate with the quantity of protein detected, and used the areas measured as reflecting protein quantity. Normalization involved dividing the area by that obtained for tubulin 4 from that sample,, which served as the housekeeping standard.

### Supplementary methods

Immunohistochemistry, confocal and electron microscopy, fluorescent in situ hybridization (FISH), cell cultures, siRNA design and production and AChE activity tests were as described [45] (and/or are all detailed under Supplementary Methods (Text S1).

Real Time PCR, TUNEL Apoptosis assay, immunocytochemistry, protein extraction were as described [46] (Text S1, Table S3 antibodies list and Table S4 probes list).

## Supporting Information

Text S1 Supplementary Materials and Methods.
(0.05 MB DOC)

Figure S1 N-AChE-S characteristics. A. Cellular and secreted AChE forms. Hydrolytic activity of the AChE variants in transiently transfected U87MG cell extracts and medium. Note that AChE-R and N-AChE-R are largely secreted, whereas AChE-S and N-AChE-S are largely cellular. B. Immunoblot and activity staining. Top: Immunoblot. Note low-level expression of N-AChE-S and N-AChE-R compared to the corresponding shorter counterparts. Bottom: Activity staining in a native gel. Note migration and staining intensity differences. C. Km analysis. Percentage of maximal acetylthiocholine (ATCh) hydrolytic activity as a function of substrate concentration. Note indistinguishable Km values (0.33±0.092, 0.29±0.035, 0.35±0.13, and 0.39±0.17) and similar Hill's coefficients (0.94±0.12, 0.85±0.05, 0.91±0.17, 0.87±0.18) for the AChE-R, AChE-S, N-AChE-R and N-AChE-S variants, respectively. D. Substrate inhibition. All variants showed inhibition under acetylthiocholine (ATCh) concentrations >5 mM.
(1.42 MB TIF)

Figure S2 N-AChE-S Sustained Unfolded protein Response under Transfection. A. GRP78 levels: Immunoblot shows similar levels of GRP78, an unfolded protein response marker, in cells co-transfected with AChE-S, N-AChE-R and N-AChE-S. B. XBP splicing ratios: 3% Agarose gel shows the PCR products of XBP transcripts, the upper band is the unspliced form, and the lower band is the spliced form. N-AChE-S transfection sustained a similar ratios between unspliced/spliced forms to those seen with the other AChE variants. Thapsigargin induced ER stress (below) showed higher expression of XBP and a higher ratio for the spliced form (columns).
(1.30 MB TIF)

Figure S3 Membranal localization of N-AChE-S. Top Scheme: Antibodies to the N-terminus or core domain of N-AChE-S (NS). SP: Original signal peptide. A. Electron micrographs: Gold beads-decorated antibodies labeling of U87MG cells transfected with N-AChE-S. Note labeling close to the plasma membrane or within endocytotic, clathrin-coated vesicles (arrows). B. Labeling distribution. Columns: Distance of gold beads from the plasma membrane. 11% of intracellular N-AChE-S labeling for both the N-terminus and the core domain occurred close to the plasma membrane, another 11% - in endocytotic vesicles.
(1.86 MB TIF)

Table S1 (0.07 MB DOC)

Table S2 (0.04 MB DOC)

Table S3 (0.04 MB DOC)

Table S4 (0.03 MB DOC)
