# Supplementary material for: N-Acetylcholinesterase-Induced Apoptosis in Alzheimer's Disease
Source: PLoS One. 2008 Sep 1;3(9):e3108. doi: 10.1371/journal.pone.0003108 (PMC2518620; doi:10.1371/journal.pone.0003108)
Supplement: Text S1 — Supplementary Materials and Methods. (0.05 MB DOC) [file pone.0003108.s001.doc]

**Text S1**

### **Supplementary Materials and Methods**

RNA extraction and real-time RT-PCR: The RNeasy kit (Qiagen, Valencia, CA) was used as per manufacturer’s instructions for RNA extraction. DNase was applied to remove DNA contamination. RNA integrity was confirmed by gel electrophoresis, and RNA concentration and purity was assessed spectrophotometrically. For cDNA synthesis, 0.4 μg RNA was used for each sample (Promega, Madison, WI). Quantitative (Q)RT-PCR was performed in duplicate for each sample using ABI prism 7900HT and SYBR green master mix (Applied Biosystems, Foster City, CA). ROX, a passive reference dye, was used for signal normalization across the plate, using, ß-actin mRNA as a reference transcript. Annealing temperature was 60ºC for all primers. Serial dilution of samples served to evaluate primers efficiency and the appropriate cDNA concentration that yields linear changes. Melting curve analysis and amplicons sequencing served to verify the end product. Primers employed included: For N-AChE (E1e) (Gene Bank no. AY389979) (188+) AATGCTAGGCCTGGTGATGT and (285-) (NM_000665) GGCAGTGGAAACTTCTGGA, for β-actin (NM 001101) (+1079-1099) CACTCTTCCAGCCTTCCTTC and (-1127-1147)GGATGTCCACGTCACACTTC (Nos. denote nucleotide position in the respective sequences).

Immunohistochemistry: Paraffin slices handled simultaneously to minimize inter-slides variability were dewaxed and blocked for 1h in Tris 10% serum-blocking solution containing horse and goat serum, 5% each. Primary antibodies (Table 2) were diluted in 2% TBS-milk /5% serum mix and applied for 2h at room temp or overnight at 4°C. Corresponding biotin-conjugated secondary antibodies were used. For anti AChE-S (C16, Santa Cruz, CA, USA) or the extended N-terminus (in-house antibodies); detection was with the ABC kit (Vector) with 3,3′-diaminobenzidine as substrate. Zeiss Axioplan or Bio-Rad MRC – 1024 confocal microscopy served for analysis. Labeling intensity was quantified with ImagePro Plus 4.5 (Media Cybernetics). Mann Whitney or Median test was performed for statistical significance.

Confocal Microscopy: for FISH experiments, brain slices were scanned using an FV-1000 confocal microscope (Olympus, Japan), equipped with an IX81 inverted attachment and a. A 40X/1.3 oil immersion objective. Excitation for DAPI was 405 and emission was collected using a 430-470 nm filter. Cy2 fluorescence was imaged using the 488 nm laser line for excitation and a 505-525 nm was used to collect the emission. In microinjection experiments, Cy3 excitation fluorescence was imaged using the 543 nm laser line for excitation and a 560-620 nm was used to collect the emission.

Fluorescent in Situ hybridization (FISH):

Paraffin-embedded tissue sections were subjected to de-paraffinization with xylene (two 5-min washes) followed by decreasing ethanol washes (100%, 75%, 50% and 25%) in PBT (0.13 M NaCl, 7 mM Na_2_HPO_4_·7H_2_O, 3 mM NaH_2_PO_4_·H2O, 0.1% Tween20), and finally twice with PBT. Quenching was applied to suppress autofluorescence [1]. Sections were then treated with10μg/ml proteinase K (Boehringer Mannheim, Germany) for 8 min at room temp and washed with PBT. Prehybridization (60°C for 30 min) was in 50% formamide, 5X Sodium saline citrate (SSC), 50 µg/ml yeast tRNA (Boehringer Mannheim, Germany) and 50 µg/ml heparin (Sigma St. Louis, MI) in DDW. Pre-heated hybridization mix (including biotin-conjugated probes, 10 µg/ml) was added (in a humid chamber, 90 min or over-night at 52°C for hE1e (N-AChE) and E6 (AChE-S) probes respectively). Two high-stringency washes at 60°C in solution 1 (50% formamide, 5X SSC pH 4.5, 0.5% sodium dodecyl sulfate in DDW), and 2 (50% formamide, 5X SSC pH 4.5 in DDW) were followed by two washes in TBST (25 mM Tris-HCl pH 7.5, 136 mM NaCl, 2.7 mM KCl, 0.05% Tween20), and incubation with 1% skim milk in TBST to prevent non-specific streptavidin interactions. Detection involved 40 min incubation with streptavidin-conjugated Cy3 (Jackson, West Grove, PA, USA) diluted 1:100, at room temp. Slides were then washed 3 times with TBST, once in DDW, and mounted with ImmunoMount (Shandon, Pittsburgh, PA). All slides were handled simultaneously to minimize inter-slides variability. Negative control slides were incubated with a zeta-globin probe that is not expressed in the adult brain. The 2’O-methyl, 5'-Biotinylated cRNA probes (Microsynth, Balgach, Switzerland) were as in Supplementary Table 4:

Immunoblots. SDS gels were prepared (10 to 15% acrylamide, depending on protein size). Blocking of phosphorylated proteins was performed using 5% BSA in TBST, while for non-phosphorylated proteins we used 5% skim milk. (Presented are representative blots of 3 experiments).

TUNEL Apoptosis assay: Terminal UTP-transferase nick-end labeling (TUNEL) was performed using the Apo Alert kit from Clontech (Palo Alto, CA). Cell numbers for each experiment ranged at 3000-11000. Cell counts were determined using a Zeiss Axiophot microscope (magnification 400x). Average ±S.E.M. values (percentage of positive cells) were counted in 2-3 independent experiments (each performed in duplicates) in 8 fields on each cover slip.

Immunocytochemistry: Cells were grown on cover-slips coated with poly-L-lysine. Medium was removed 24h post-transfection, cells were washed with phosphate-buffered saline ((PBS): KH_2_PO_4_ 0.1M; pH=7.4, 0.9% NaCl), fixed in fresh 4% paraformaldehyde in PBS (40 min, room temp), washed and maintained in PBS at 4°C. Fixed cells were incubated with 100 mM glycine (5 min x 3), followed by a PBS wash, incubation (1h, room temp) in a blocking buffer (5% BSA, 0,8% Triton X-100 in PBS) and then overnight at 4°C with polyclonal antibodies (Table 2). Detection involved incubation with Biotin-conjugated secondary antibodies (Jackson, PA, USA), in blocking buffer at a concentration of 1:10,000 (1h, room temp), 3 washes (PBS, 5 min), incubation in Cy3-conjugated streptavidin in PBS, wash and DAPI labeling for nuclear staining. Tissue sections were cover-slipped in Shandon immunomount (Shandon, PA, USA) and were analyzed by fluorescence microscopy using a Zeiss Axiophot microscope equipped with a digital camera. Average ± Standard error of the mean (S.E.M). density values were calculated in 2-3 independent experiments (each in duplicates) in 8 fields on each cover slip.

AChE activities: Acetylthiocholine (ACTh) hydrolysis rates were measured as detailed [2]. Readings at 405 nm were repeated at 1-min intervals for 20 min. Non-enzymatic hydrolysis of substrate was subtracted from the total rate of hydrolysis. Enzyme activity was calculated using the molar extinction coefficient for 5-thio-2-nitrobenzoate (13,600 M^-1^ cm^-1^) [3].

Cell cultures: Tissue culture reagents were from Biological Industries (Beit HaEmek, Israel). Chinese hamster ovary (CHO), human glioblastoma (U87MG), human embryonic kidney (HEK293) and NIH/3T3 fibroblast cells are all grown in a humidified atmosphere with 5% CO_2_ at 37°C in Dulbecco’s modified Eagle’s medium (DMEM, Biological Industries) supplemented with 2 mM L-glutamine, 100 U/ml penicillin, 0.1 mg/ml streptomycin and 10% FCS for U87MG, CHO and 293 cells. Human fibroblast T84 and SH-SY5Y neuroblastoma cells were cultured at 37°C in a humid 5% CO2 atmosphere, in a 1:1 mixture of Eagle’s minimum essential medium and F12 medium, containing 10% fetal calf serum (FCS) and a mixture of 1% penicillin/streptomycin/amphotericin. Transfection with S, R, NS and NR plasmids was performed with lipofectamin 2000 (Invitrogen, Carlsbad, CA) as per the manufacturer’s instructions.

Electron Microscopy: Pre-embedding and immunogold staining: U87MG-cells were grown 24 hrs after transfection on Aclar sheets (Pelco International Clovis, CA) coated with poly L-lysine. Fixation was in 4% formaldehyde, 2% picric acid, 0.01% Glutaraldehyde (Agar Scientific Limited Essex, CM) and 0.1M Sodium Cacodylate trihydrate (ElectronMicroscopy Sciences, Hatfield, PA), pH 7.4 for 1hr at RT. Cryoptection and freeze and thaw.

Slides were in Blocking buffer (Egg Albumin, 2% 0.5% glycine, 0.5% lysine and 0.13% sodium azide) for 1hr.

Antibody concentration was 1:50 in 1% Egg Albumin, triton 0.01% buffer incubated for 24-48 hrs at 4°C. Secondary antibodies were from Jackson Immuno Research (goat anti-rabbit and donkey anti-goat conjugated to 6nm gold particles).

Samples were fixed, osmicated, dehydrated and embedded in Agar 100 resin (Agar Scientific, Essex, England). Blocks were sectioned using an LKB-3 ultramicrotome (LKB Instruments, Inc., Rockville, Maryland). Thin sections were stained with saturated aqueous uranyl acetate and lead citrate solutions and observed with Tecnai 12 (Phillips, Eindhoven, The Netherlands) Transmission Electron Microscope equipped with MegaView II CCD camera and AnalySIS® version 3.0 software (SoftImaging System GmbH,. Münster, Germany).

Total protein extraction: Homogenates were diluted in Low Salt detergent, kept on ice for 1h; centrifuged in a table centrifuge at maximum speed (13000 rpm) for 30 min 4ºC, and supernatant transferred to a clean tube and kept frozen at -70°C until use. Differential extraction was described. [4].

siRNA design: siRNAs were designed as instructed in the algorithm by Cenix BioScience at the Ambion website ([www.ambion.com](http://www.ambion.com/)). Numbers denote the position of the first siRNA nucleotide on the gene sequence. The AChE siRNA used was 1024, AACGCGGGAGACTTCCACGGC. Additional siRNAs designed to serve as controls were computer generated and then compared to the different AChE sequences, to ensure that the controls did not match the genomic sequences. The control sequence used was: AAGTCCTACCAAGCATCGGAC.

SiRNA Synthesis: siRNAs were synthesized using the "Silencer® siRNA Construction Kit" (Ambion, Tel Aviv, Israel), essentially according to the manufacturer’s manual. The oligonucleotide templates were purchased from Sigma (Jerusalem, Israel).

Success of the synthesis was confirmed by a 2.5% agarose gel electrophoresis.

The concentration of each siRNA was determined using The NanoDrop® ND-1000 UV-Vis Spectrophotometer (Nanodrop Technologies, Wilmington, DE, USA).

Transfection was performed according to manufacturer's instructions (Lipofectamine™ 2000 Stealth/siRNA transfection, Invitrogen, Carlsbad, CA).

## References

1. Sun A, Nguyen XV, Bing G (2002) Comparative analysis of an improved thioflavin-s stain, Gallyas silver stain, and immunohistochemistry for neurofibrillary tangle demonstration on the same sections. J Histochem Cytochem 50: 463-472.

2. Diamant S, Podoly E, Friedler A, Ligumsky H, Livnah O, et al. (2006) Butyrylcholinesterase attenuates amyloid fibril formation in vitro. Proc Natl Acad Sci U S A 103: 8628-8633.

3. Ellman GL, Courtney D, Andres VJ, Featherstone RM (1961) A new and rapid colorimetric determination of acetylcholinesterase activity. Biochem Pharmacol 7: 88-95.

4. Soreq H, Seidman S (2001) Acetylcholinesterase--new roles for an old actor. Nat Rev Neurosci 2: 294-302.
