# Supplementary figures and images for: N-Acetylcholinesterase-Induced Apoptosis in Alzheimer's Disease
Source: PLoS One. 2008 Sep 1;3(9):e3108. doi: 10.1371/journal.pone.0003108 (PMC2518620; doi:10.1371/journal.pone.0003108)

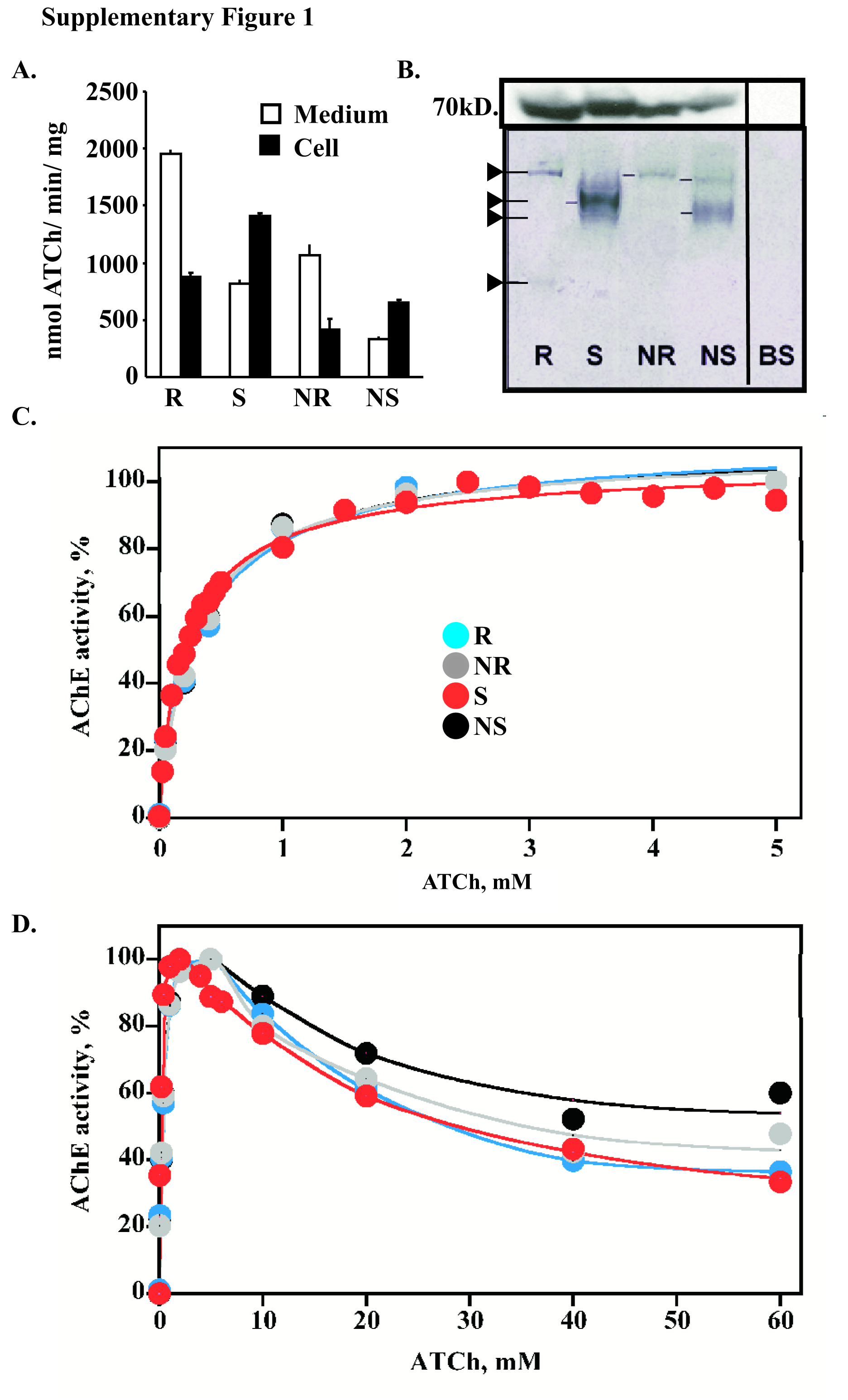

Supplement: Figure S1 — N-AChE-S characteristics. A. Cellular and secreted AChE forms. Hydrolytic activity of the AChE variants in transiently transfected U87MG cell extracts and medium. Note that AChE-R and N-AChE-R are largely secreted, whereas AChE-S and N-AChE-S are largely cellular. B. Immunoblot and activity staining. Top: Immunoblot. Note low-level expression of N-AChE-S and N-AChE-R compared to the corresponding shorter counterparts. Bottom: Activity staining in a native gel. Note migration and staining intensity differences. C. Km analysis. Percentage of maximal acetylthiocholine (ATCh) hydrolytic activity as a function of substrate concentration. Note indistinguishable Km values (0.33±0.092, 0.29±0.035, 0.35±0.13, and 0.39±0.17) and similar Hill's coefficients (0.94±0.12, 0.85±0.05, 0.91±0.17, 0.87±0.18) for the AChE-R, AChE-S, N-AChE-R and N-AChE-S variants, respectively. D. Substrate inhibition. All variants showed inhibition under acetylthiocholine (ATCh) concentrations >5 mM. (1.42 MB TIF) [file pone.0003108.s002.tif]

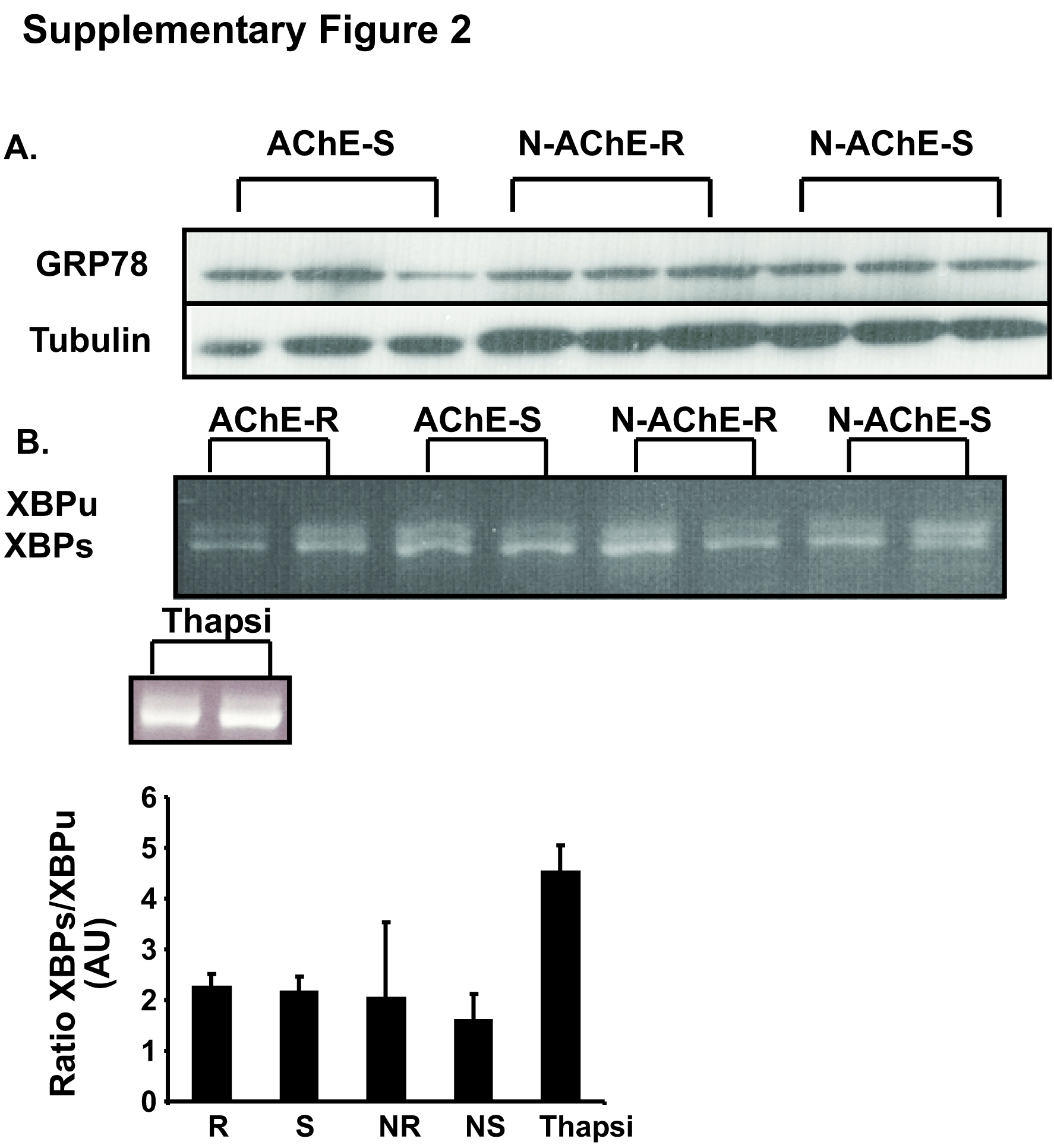

Supplement: Figure S2 — N-AChE-S Sustained Unfolded protein Response under Transfection. A. GRP78 levels: Immunoblot shows similar levels of GRP78, an unfolded protein response marker, in cells co-transfected with AChE-S, N-AChE-R and N-AChE-S. B. XBP splicing ratios: 3% Agarose gel shows the PCR products of XBP transcripts, the upper band is the unspliced form, and the lower band is the spliced form. N-AChE-S transfection sustained a similar ratios between unspliced/spliced forms to those seen with the other AChE variants. Thapsigargin induced ER stress (below) showed higher expression of XBP and a higher ratio for the spliced form (columns). (1.30 MB TIF) [file pone.0003108.s003.tif]

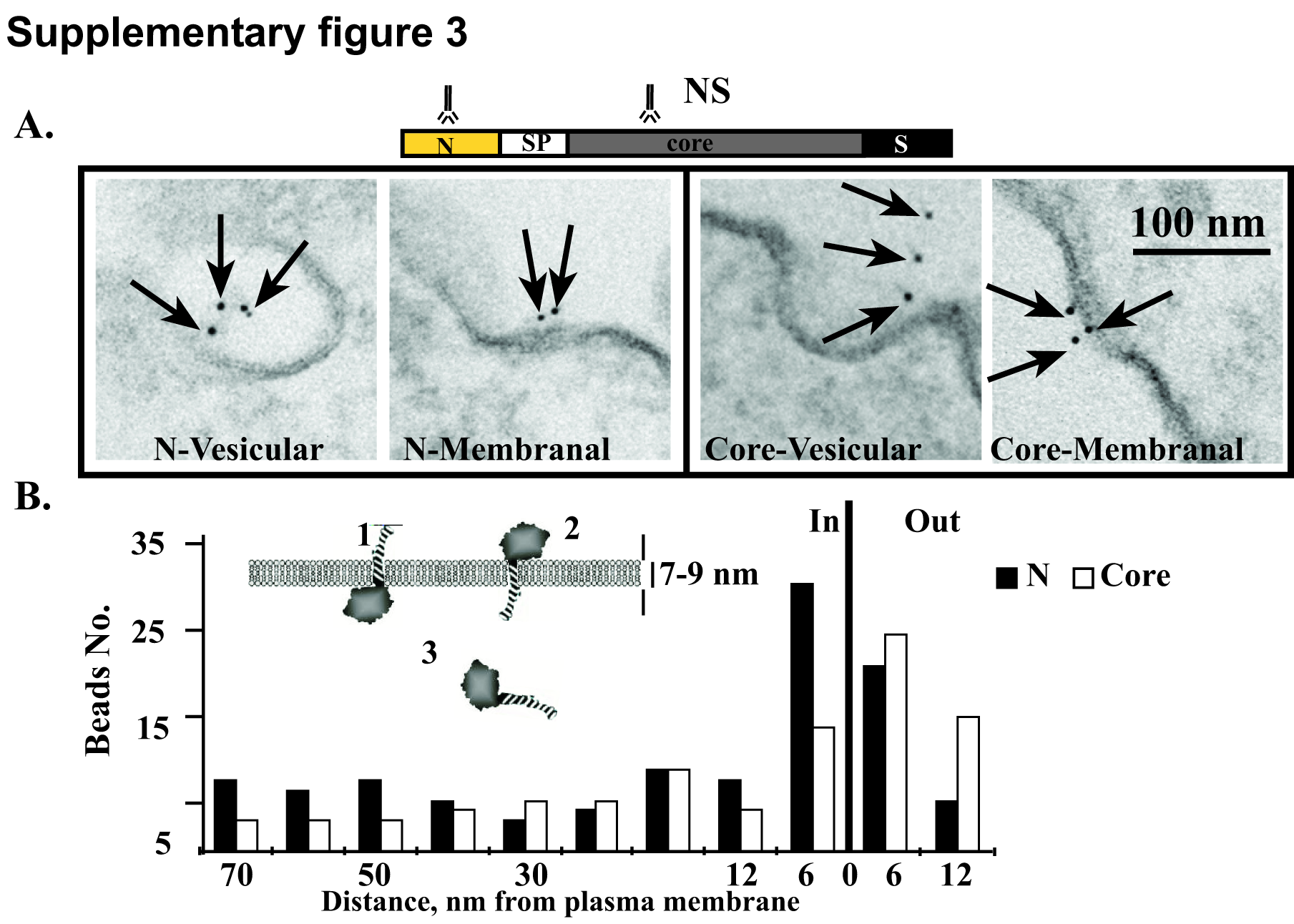

Supplement: Figure S3 — Membranal localization of N-AChE-S. Top Scheme: Antibodies to the N-terminus or core domain of N-AChE-S (NS). SP: Original signal peptide. A. Electron micrographs: Gold beads-decorated antibodies labeling of U87MG cells transfected with N-AChE-S. Note labeling close to the plasma membrane or within endocytotic, clathrin-coated vesicles (arrows). B. Labeling distribution. Columns: Distance of gold beads from the plasma membrane. 11% of intracellular N-AChE-S labeling for both the N-terminus and the core domain occurred close to the plasma membrane, another 11% - in endocytotic vesicles. (1.86 MB TIF) [file pone.0003108.s004.tif]
