# Supplementary material for: N-Acetylcholinesterase-Induced Apoptosis in Alzheimer's Disease
Source: PLoS One. 2008 Sep 1;3(9):e3108. doi: 10.1371/journal.pone.0003108 (PMC2518620; doi:10.1371/journal.pone.0003108)
Supplement: Table S1 — (0.07 MB DOC) [file pone.0003108.s005.doc]

**Table S**1

| **TUNEL** |  | |  |  |  | |  | |
| --- | --- | --- | --- | --- | --- | --- | --- | --- |
| **transfected cells** | | |  | **medium of transfected cells** | | | |  |
| **293** | | **Fold** | **SEM** | **Fold** | **SEM** | |  | |
| control | | 1.00 | 0.33 | 0.98 | 0.49 | |  | |
| AChE-R | | 1.41 | 0.38 | 0.44 | 0.31 | |  | |
| AChE-S | | 1.48 | 0.39 | 0.57 | 0.32 | |  | |
| N-AChE-R | | 1.78 | 0.34 | 0.57 | 0.43 | |  | |
| N-AChE-S | | 4.07 | 0.38 | 1.01 | 0.46 | |  | |
| ttest | | ctrx R | 0.422501 | ctrx R | 0.963 | |  | |
|  | | Ctrx S | 0.364204 | Ctrx S | 0.326 | |  | |
|  | | CtrxNR | 0.123516 | Ctrx NR | 0.450 | |  | |
|  | | ctrx NS | 3.04E-05 | ctrx NS | 0.361 | |  | |
| **U87MG** | |  |  |  |  | |  | |
| control | | 1.00 | 0.66 | 0.74 | 0.09 | |  | |
| AChE-R | | 1.03 | 1.59 | 0.84 | 0.07 | |  | |
| AChE-S | | 1.84 | 0.70 | 0.79 | 0.19 | |  | |
| N-AChE-R | | 1.84 | 0.92 | 0.95 | 0.18 | |  | |
| N-AChE-S | | 15.84 | 2.83 | 0.99 | 0.16 | |  | |
| ttest | | ctrx R | 0.259155 | ctrx R | 0.209 | |  | |
|  | | Ctrx S | 0.974725 | Ctrx S | 0.430 | |  | |
|  | | CtrxNR | 0.468494 | Ctrx NR | 0.436 | |  | |
|  | | ctrx NS | 0.001021 | ctrx NS | 0.887 | |  | |
| **CHO** | |  |  |  |  | |  | |
| control | | 1.00 | 0.43 | 0.98 | 0.21 | |  | |
| AChE-R | | 4.49 | 1.52 | 0.65 | 0.07 | |  | |
| AChE-S | | 2.84 | 1.28 | 0.92 | 0.11 | |  | |
| N-AChE-R | | 3.11 | 1.33 | 0.71 | 0.13 | |  | |
| N-AChE-S | | 12.73 | 1.34 | 1.00 | 0.18 | |  | |
| ttest | | ctrx R | 0.086214 | ctrx R | 0.932 | |  | |
|  | | Ctrx S | 0.206076 | Ctrx S | 0.107 | |  | |
|  | | CtrxNR | 0.170948 | Ctrx NR | 0.705 | |  | |
|  | | ctrx NS | 0.000262 | ctrx NS | 0.217 | |  | |
| **T84** | |  |  |  | |  | | |
| control | | 1.00 | 1.00 |  | | |
| AChE-R | | 3.95 | 2.14 |  | | |
| AChE-S | | 5.77 | 3.08 |  | | |
| N-AChE-R | | 5.29 | 2.04 |  | | |
| N-AChE-S | | 20.84 | 2.18 |  | | |
| ttest | | ctrx R | 0.240094 |  | | |
|  | | Ctrx S | 0.17716 |  | | |
|  | | CtrxNR | 0.087434 |  | | |
|  | | ctrx NS | 9.92E-06 |  | | |
