# Supplementary material for: N-Acetylcholinesterase-Induced Apoptosis in Alzheimer's Disease
Source: PLoS One. 2008 Sep 1;3(9):e3108. doi: 10.1371/journal.pone.0003108 (PMC2518620; doi:10.1371/journal.pone.0003108)
Supplement: Table S2 — (0.04 MB DOC) [file pone.0003108.s006.doc]

**Table S2**

**Patient details, Tau and N-AChE levels^1^**

| **Patient (Gender; age)** | **Diagnosis (PMD)** | **N-AChE** | **Tau-P** | **hE1e** |
| --- | --- | --- | --- | --- |
| 02-005 (f;42) | AD, Braak 6 (06:10) | 57.053 | 52.03 | 36.58 |
| 00-025 (f;41) | NDC, AD, Braak 0 (13:30) | 32.8 | Negative | 6.8 |
| 02-079 (f;58) | AD (04:30) | 63.17 | 60.54 | ND |
| 99-067 (f;59) | NDC, AD, Braak 1 (06:20) | 40 | Negative | 6.68 |
| 03-009 (m;65) | AD, Braak 5 (07:20) | 41.44 | 30.15 | ND |
| 02-099 (m;69) | AD, Braak 6 (05:30) | 60.80 | 64.12 | ND |
| 00-017 (f;72) | NDC, AD, Braak 1 (06:45) | 33.9 | Negative | 6.23 |
| 02-061 (f;76) | AD, Braak 5 (10:45) | ND | ND | 19.2 |
| 02-088 (f;78) | AD, Braak 5 (04:00) | 42.39 | 53.61 | ND |
| 02-085 (f;79) | AD, Braak 5 (04:15) | 55.72 | 73.5 | ND |
| 01-021 (m;82) | NDC, AD, Braak 1 (07:40) | 40.3 | Negative | 18.28 |
| 02-056 (f;85) | AD, Braak 5 (03:45) | 36.36 | 18.32 | 20.04 |
| 02-080 (f;86) | AD, Braak 5 (04:10) | 45.67 | 63.55 | ND |
| 02-102 (f;88) | AD, Braak 5 (03:15) | 48.96 | 45.81 | 28.23 |

**^1^**Shown are patient numbers, gender, age, Braak stage [1] and post-mortem delay (PMD) in hrs. Labeling density for N-AChE and Tau-P reflects immunohistochemistry results, hE1e labeling displays FISH data. NDC, non-demented control. ND, not determined.

**References**

1. Braak H, Braak E (1991) Neuropathological stageing of Alzheimer-related changes. Acta Neuropathol (Berl) 82: 239-259.
