# Supplementary material for: N-Acetylcholinesterase-Induced Apoptosis in Alzheimer's Disease
Source: PLoS One. 2008 Sep 1;3(9):e3108. doi: 10.1371/journal.pone.0003108 (PMC2518620; doi:10.1371/journal.pone.0003108)
Supplement: Table S3 — (0.04 MB DOC) [file pone.0003108.s007.doc]

**Table S3**

**Antibodies**

| **Antibody** | **Company** | **Dilution used** |
| --- | --- | --- |
| Activated caspase-3 | *Cell Signaling | 1:100 IB |
| Activated caspase-9 | *Cell Signaling | 1:100 IB |
| Bcl-2 | *Cell Signaling | 1:100 IB |
| AChE N19 | ** BD PharMingen | 1:100 IB |
| Bax (ab7514) | ******* abcam | 1:100 IB 1:1000 WB |
| AChE-S (C16) | # Santa Cruz | 1:100 IB |
| N-AChE | ## Eurogentec | 1:100 IB 1:1000 WB |
| Tubulin α4 | # Santa Cruz | 1:1000 WB |
| GSK3-β(l-17) | # Santa Cruz | 1:1000 WB |
| P-GSK-3α/β(Ser21/9) | *Cell Signaling | 1:1000 WB |
| AChE (N19), sc-8257 | # Santa Cruz | 1:1000 WB |
| GSK3α/β(P-Y216+Y279) | ******* abcam | 1:1000 WB |
| Tau-P (AT-8) | $ INNOGENETICS | 5:1000 IB 10:1000 WB |
| GRP78 (#3183) | *Cell Signaling | 1:2000 WB |

IB- Immunoblot, WB- Westernblot.

1C16 peptide # Santa Cruz,

2 N-AChE peptides: KVRSHPSGNQHRPTRG GSRSFHCRRGVRPRPA ## Eurogentec.

*Cell Signaling Technology Inc (Danvers, MA)

** BD PharMingen (Becton-Dickinson, Oxford, UK).

***abcam (Cambridge, UK).

# Santa Cruz (Santa Cruz, CA).

## Eurogentec, (Seraing, Belgium).

$ Technologiepark (Gent, Belgium)
