# Supplementary material for: N-Acetylcholinesterase-Induced Apoptosis in Alzheimer's Disease
Source: PLoS One. 2008 Sep 1;3(9):e3108. doi: 10.1371/journal.pone.0003108 (PMC2518620; doi:10.1371/journal.pone.0003108)
Supplement: Table S4 — (0.03 MB DOC) [file pone.0003108.s008.doc]

**Table S4.**

**Probes employed for FISH analyses**

| Probe | Accession # | Sequence 5’-3’ | Position |
| --- | --- | --- | --- |
| hE6 | NM_000665 | ccgggggacgucggggugggguggggaugggcagagucuggggcucgucu | 2022-2071 |
| hE1e | AY389977 | ucgucaccaggguccggucggggcaugacaucaccaggccuagca | 227-271 |
| hζglobin | BC027892 | tgatggtcctctcagtcttggtcagagacatggcggcagggtgggcagct | 17-66 |

Position denotes nucleotides no.
